# Injection of amyloid-β to lateral ventricle induces gut microbiota dysbiosis in association with inhibition of cholinergic anti-inflammatory pathways in Alzheimer’s disease

**DOI:** 10.1186/s12974-022-02599-4

**Published:** 2022-09-28

**Authors:** Xiao-hang Qian, Xiao-li Liu, Guang Chen, Sheng-di Chen, Hui-dong Tang

**Affiliations:** 1 Department of Neurology and Institute of Neurology, Rui Jin Hospital, Shanghai Jiao Tong University School of Medicine, Shanghai, 200025 China; 2 Department of Neurology, Shanghai Fengxian District Central Hospital, Shanghai Jiao Tong University Affiliated Sixth People’s Hospital South Campus, Shanghai, 201406 China; 3 The Second Hospital of Anhui Medical University, Anhui, 230601 China; 4 Medical Center on Aging of Ruijin Hospital Shanghai Jiao Tong University School of Medicine, Shanghai, 200025 China

**Keywords:** Alzheimer’s disease, Aβ, Gut microbiota, Cholinergic anti-inflammatory pathway

## Abstract

**Background:**

Alzheimer's disease (AD) is the most common neurodegenerative disease and its pathogenesis is still unclear. There is dysbiosis of gut microbiota in AD patients. More importantly, dysbiosis of the gut microbiota has been observed not only in AD patients, but also in patients with mild cognitive impairment (MCI). However, the mechanism of gut microbiota dysbiosis in AD is poorly understood. Cholinergic anti-inflammatory pathway is an important pathway for the central nervous system (CNS) regulation of peripheral immune homeostasis, especially in the gut. Therefore, we speculated that dysfunction of cholinergic anti-inflammatory pathway is a potential pathway for dysbiosis of the gut microbiota in AD.

**Methods:**

In this study, we constructed AD model mice by injecting Aβ_1–42_ into the lateral ventricle, and detected the cognitive level of mice by the Morris water maze test. In addition, 16S rDNA high-throughput analysis was used to detect the gut microbiota abundance of each group at baseline, 2 weeks and 4 weeks after surgery. Furthermore, immunofluorescence and western blot were used to detect alteration of intestinal structure of mice, cholinergic anti-inflammatory pathway, and APP process of brain and colon in each group.

**Results:**

Aβ_1–42_ i.c.v induced cognitive impairment and neuron damage in the brain of  mice. At the same time, Aβ_1–42_ i.c.v induced alteration of gut microbiota at 4 weeks after surgery, while there was no difference at the baseline and 2 weeks after surgery. In addition, changes in colon structure and increased levels of pro-inflammatory factors were detected in Aβ_1–42_ treatment group, accompanied by inhibition of cholinergic anti-inflammatory pathways. Amyloidogenic pathways in both the brain and colon were accelerated in Aβ_1–42_ treatment group.

**Conclusions:**

The present findings suggested that Aβ in the CNS can induce gut microbiota dysbiosis, alter intestinal structure and accelerate the amyloidogenic pathways, which were related to inhibiting cholinergic anti-inflammatory pathways.

**Supplementary Information:**

The online version contains supplementary material available at 10.1186/s12974-022-02599-4.

## Background

Alzheimer’s disease (AD) is the most common neurodegenerative disorder, accounting for 60%–80% dementia. The typical pathological change of AD are extracellular plaques composed of amyloid-β (Aβ) peptide and intracellular neurofibrillary tangles composed of hyperphosphorylated tau protein [1]. Until now, a serious studies have demonstrated the bidirectional, constant communication between the central nervous system (CNS) and the gastrointestinal tract through the brain-gut-microbiota axis in neurodegenerative disorder, such as Parkinson's disease, and also including the AD [2, 3]. On the one hand, alteration of gut microbiome has been reported in both the patients and mouse model of AD [4]. What’s more, the similar alteration as AD in gut microbiota has been observed in mild cognitive impairment (MCI) patients, which is considered to be a transitional phase between normal cognitive function and AD [5, 6]. This provides a new idea for early diagnosis of AD through the excrement. In addition, the change of enteric nervous system and multiple immune cell types in the gut were also reported in AD mouse model [7, 8]. On the other hand, altering the structure of gut microbiota can ameliorate the pathology, neuroinflammation and cognitive impairment through transfer of a healthy microbiota or antibiotic-induced perturbations [9, 10]. Furthermore, Aβ amyloid pathology was reduced in germ-free APP/PS1 transgenic mice [11]. However, how the CNS and gut microbiota interplay is still a mystery.

The vagus nerve, composed of 20% efferent and 80% afferent fibers, is the 10th cranial nerve. It is the fastest and most direct route that connects the gut and brain [3]. More importantly, the vagus nerve is widely distributed in abdominal organs, including moving caudally from the proximal duodenum to the level of the transverse colon [12]. Therefore, the vagus nerve plays a key role in the CNS and gut microbiota interplay. For example, the cholinergic anti-inflammatory pathway, transmitting the neural signal through the vagus nerve, plays an important role in modulating circulation levels of proinflammatory cytokines [13]. Altering parasympathetic activity has been proved to relate to the bacterial overgrowth, bacterial translocation or even functional bowel disorders [14, 15]. Despite considerable importance of the cholinergic anti-inflammatory pathway, there is little known about the molecular basis for central regulation of the cholinergic anti-inflammatory pathway based on the vagus nerve. In 2006, Valentin et al. demonstrated that the central muscarinic cholinergic receptor can regulate the activation of the cholinergic anti-inflammatory pathway to influence systemic inflammatory response [16]. Three years later, Valentin et al. proved the central acetylcholinesterase activity controls systemic cytokine levels through the cholinergic anti-inflammatory pathway [17]. However, the central cholinergic system has been implicated in learning and memory of brain, and dysregulation of cholinergic neurotransmission can contribute to the symptoms of AD [18, 19]. Moreover, some studies have proved that Aβ can induce a significant reduction in mM1 and M2 mAChR in some brain area, such as hippocampus, the medial septum-diagonal band of Broca (MS-nDBB) complex [20, 21]. Therefore, it is reasonable to speculate that in AD, Aβ regulates the function of the vagal cholinergic anti-inflammatory pathway by affecting the expression and survival of central cholinergic neurons, thereby affecting the intestinal homeostasis, leading to the destruction of intestinal mucosal barrier, the change and displacement of gut microbiota, and ultimately accelerating the progression of AD.

In this study, we constructed AD model mice by intracerebroventricular injecting Aβ_1–42_, and detect the abundance of gut microbiota and structure of gut after injection. On this basis, we explored the role of vagal cholinergic anti-inflammatory pathway regulation of gut microbiota, intestinal function and neural circuits regulated by the upstream the CNS in AD mouse models, providing theoretical basis for early diagnosis of AD through gut microbiota and intestinal function.

## Methods

### Animals

**Fig. 1:**
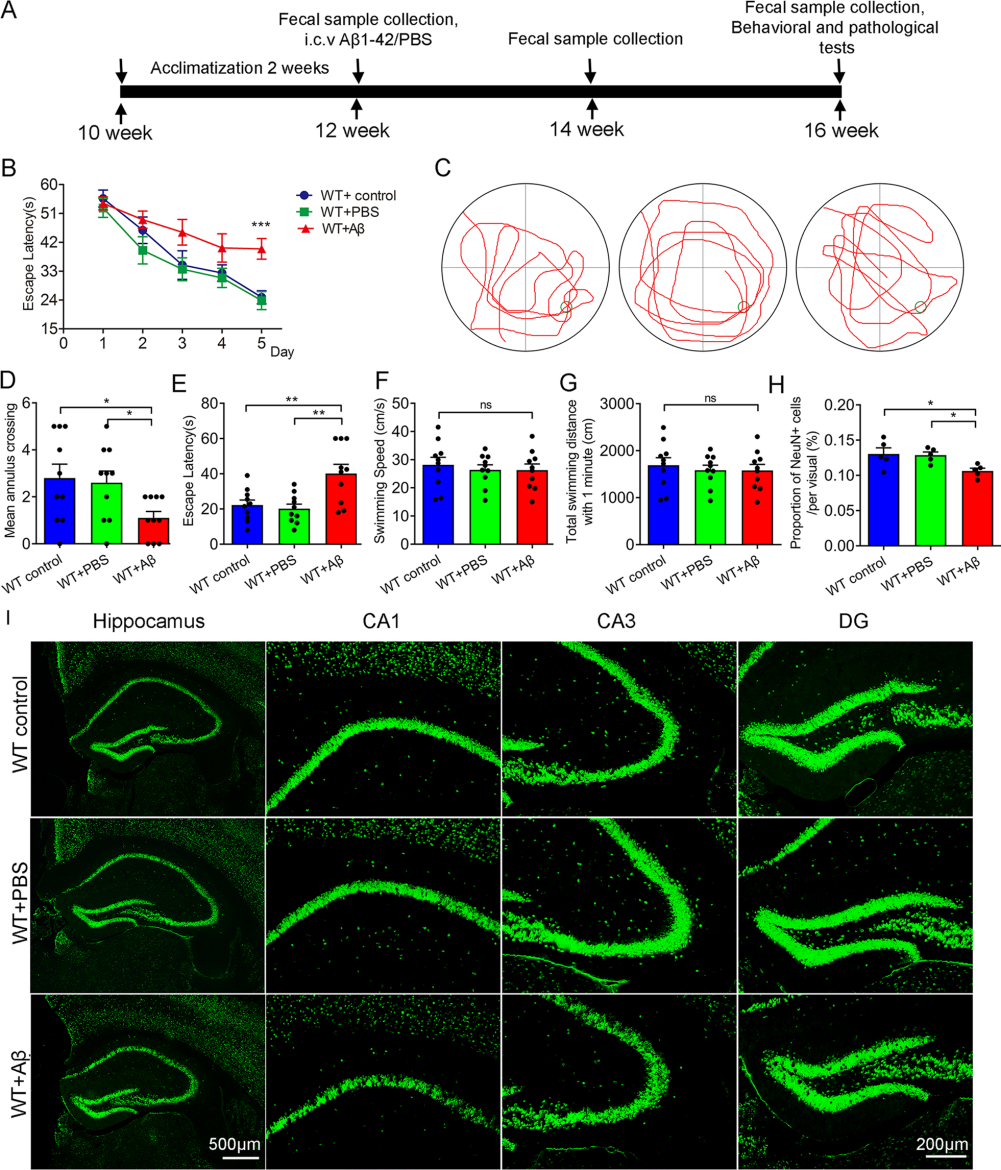
Aβ_1–42_ i.c.v-induced cognitive impairment and neuronal impairment in mice. **A** Experimental design. Male C57BL/6 J mice aged 10 weeks were fed for 2 weeks under standard laboratory conditions to acclimate to the environment. At 12 weeks of age, mice were randomly divided into three groups: control group (WT control), sham operation group (WT + PBS), and Aβ_1–42_ i.c.v group (WT + Aβ). After fecal samples were collected from each group at baseline, surgical administration was performed. At 2 weeks after surgery, feces samples were collected. After 4 weeks, feces samples were collected, and behavioral and pathological detects were performed. **B** The escape latency from the day1 to day 5. **C** The swimming track to find the platform on the day 6. **D** Platform crossing times within 1 min on the day 6. **E** The escape latency within 1 min on the day 6. **F** The swimming speed  within 1 min. **G** The swimming distance  within 1 min. n = 10 per group. **H**–**I** Immunofluorescence and quantification of NeuN in hippocampus (Scale bars, 500 μm) and CA1, CA3, and DG region (Scale bars, 200 μm) of each group (n = 5 per group). Data were presented as mean ± SEM. Statistics were analyzed using one-way analysis of variance (ANOVA) and followed by Fisher’s least significant difference (LSD) test. ∗ P < 0.05, **P < 0.01, ns: no statistical difference

Male C57BL/6J mice aged 10 weeks were generated by Shanghai Model Organisms Center (Shanghai, China). The mice were raised in individual ventilation cage (specific pathogen free, five mice per cage) with standard laboratory conditions (room temperature: 22 ± 2 ℃, relative humidity at 55 ± 5%, 12:12 h light–dark cycle). All procedures involving the animals were conducted according to the Institutional Guidelines and associated guidelines in the European Communities Council (86/609/ ECC). The experimental protocol was approved by the Animal Ethics Committee of school of Shanghai Model Organisms Center (authorization number: 2022-0008). After 2 weeks of acclimatization, the mice were randomly divided into 3 groups: the control group, the sham-operated group, and the Aβ intracerebroventricular injection (i.c.v) group, with 10 mice in each group. The flowchart of this study is shown in Fig. 1A.

### Establishment of Alzheimer’s disease model

The establishment of AD mouse model by injecting Aβ_1–42_ into the lateral ventricle has been adopted by many studies [22, 23]. Aβ_1–42_ oligomers (Sigma-Aldrich, Catalog Number: A9810) was prepared as described previously [22]. Briefly, Aβ_1–42_ was dissolved in 0.1 M phosphate-buffered saline (PBS) to a final concentration of 1 mg/ml. Then, the Aβ_1–42_ solution was placed at 37 °C for 4 days to form Aβ_1–42_ oligomers and stored at − 20 °C. Finally, the Aβ_1–42_ oligomers (400 pmol/mouse) was administered by intracerebroventricular injected (i.c.v) through a microsyringe (Hamilton) [22–24]. After peritoneal injection of sodium pentobarbital (0.067 mg/g), mice in experimental group and sham-operated group were injected with 3 μl Aβ_1–42_ oligomers solution or PBS within 10 min, respectively. After injection, the needle was left in place for 5 min and slowly withdrawn to prevent reflux of liquid. The coordinates previously referenced by bregma are mediolateral (ML) = 1.0 mm, anteroposterior (AP) =  − 0.1 mm, and dorsoventral =  − 3.0 mm.

### Morris water maze test

The spatial memory ability of each group was tested after surgery 4 weeks through the Morris water maze test. Morris water maze test was operated as previously described [25]. Briefly, before starting the experiment, the mice were put in a room 2 h earlier to acclimatize. Each mouse was trained 4 times per day from the four quadrants of the pool for 5 consecutive days. Each mouse was allowed to search for the platform within 60 s each time. The sixth day was the testing period, and the platform was removed from the pool. Each mouse was allowed to swim in the pool for 60 s to explore the platform position. Each mouse's trajectory in the pool was recorded by water maze software for analysis.

### Fecal sample collection and bacterial 16S rRNA sequencing and processing

Each mouse was placed in a germ-free cage to collect fecal samples within 24 h. The fecal weight of each mouse was measured within 24 h. The fecal samples was stored in freezer at − 80 °C. Each group collected 4–5 samples at 3 different time points. A total of 42 samples were collected for 16S rRNA gene amplicon sequencing. First, sample DNA extraction and purification was performed using QIAamp DNA Stool Mini Kit. The 16S rDNA V3–4 region was magnified through Applied Biosystems^®^ PCR System 9700. Primer sequences are shown below: Primer 5′–3′: 357F (5′-ACTCCTACGGRAGGCAGCAG-3′) and 806R (5′-GGACTACHVGGGTWTCTAAT-3′). After that, the products of PCR was electrophoretized using 2% agarose and purified using AxyPrepDNA gel recovery kit (AXYGEN). Then, the products were quantified using FTCFTC-3000 TM real-time PCR. Finally, the Illumina Platform was used for high-throughput sequencing of sample DNA, followed by bioinformatics analysis.

### Detection of intestinal transport function and permeability

The whole intestinal transport function was detected after surgery 4 weeks through the Evans Blue gavage [26]. After fasting for 4 h, each mouse was gavage with 100 μl 1.5% methylcellulose containing 5% Evans Blue (Sigma, E2129). After 90 min, we measured the length covered by Evans Blue and the total length of intestine, and calculated the ratio of them as the transit ability. Intestinal permeability was assessed through the fluorescein isothiocyanate [FITC]-labelled dextran method [27]. After fasting for 4 h, each mouse was given 12 mg FITC-labelled dextran (Sigma, #46944) by gavage. After 90 min, we measured the fluorescence intensity in the serum of each mouse through the multifunctional microplate meter (excitation, 490 nm; emission, 520 nm).

### Immunofluorescence assay

Mice were subjected to intracardially perfuse with precooled PBS solution and PBS solution containing 4% paraformaldehyde (PFA), respectively. After the mouse brain tissue was gently separated from the skull, it was fully fixed in PBS solution containing 4% PFA for 48 h at 4 °C. The brain was then subjected to gradient dehydration using PBS solution containing 10–30% sucrose. After OCT embedding medium (Fisher Scientific), the brain was cut into 30 μm thick. Brain slices were washed 3 times with PBS for 5 min each time. Then brain slices were soaked in PBS containing 0.5% Triton X-100 (PBS-T) for 30 min and incubated with antigen retrieval solution (Beyotime, P0090) for 5 min. After washing with PBS for 3 times again, brain slices were blocked with 10% goat serum in PBS-T for 1 h at room temperature. Brain slices were then incubated with primary antibodies overnight at 4 °C. The primary antibodies used in this study include Neu N (Abcam, ab177487), ZO-1 (ThermoFisher, PA5-85256), PGP9.5 (Abclonal, A19101), CHAT (Abclonal, A13244), and M1AChR (Santa Cruz, sc-365966), TMEM119 (abcam, ab209064). After that, PBS-T was used to wash the brain slices for 3 times and incubated with fluorescently labeled secondary antibodies and DAPI for 2 h at room temperature. Finally, brain slices were wash with PBST for 5 times, followed by sealed with anti-quench sealing tablet. Images were captured using a microscope (BX60, Olympus, Tokyo, Japan). Cell counts were manually counted. The fluorescence intensity was analyzed by Image J software.

### Western blot

After the mouse brain was removed from the skull, the hippocampus and cortex were quickly separated on ice. The brain tissue was then homogenized with lysates to extract the total protein. Total protein concentration was detected by BCA kit (ThermoFisher, USA). Total 30 μg of protein was loaded to precast SDS-polyacrylamide gels for electrophoresis, and transferred to a PVDF membrane. The PVDF membrane was blocked in 5% skim milk powder in TBST for 1 h, followed by incubating with primary antibody in 5% BSA/TBST at 4 °C overnight. The primary antibodies used in this study include ZO-1 (ThermoFisher, PA5-85256), CLDN5 (ThermoFisher, 35-2500), Synapsin1 (abcam, ab64581), PSD95 (Cell Signaling Technology, 3450), M1AChR (Santa Cruz, sc-365966), CHAT (abcam, ab178850), CHRNA7 (Abclonal, A1588), CD206 (Cell Signaling Technology, 24595), CD86 (Abclonal, A19026), NF-kb (Cell Signaling Technology, 8242), p-APP (Thr668, Cell Signaling Technology, 6986), PS1 (Cell Signaling Technology, 5643), BACE1 (Abclonal, A5266), ADAM10 (Abclonal, A10438), and β-actin (Cell Signaling Technology, 3700). After three times washed with TBST, the PVDF membrane was incubated with secondary antibodies (ThermoFisher, USA). Finally, Odyssey LI‐COR imager was used for capturing the images.

### Quantitative real-time PCR analysis

Total RNA was extracted and purified from the colon using Trizol Reagent (ThermoFisher, USA). The total RNA is then used for reverse transcription into c.DNA using a PrimerScript RT Reagent kit at 37 °C for 15 min (TaKaRa, Japan). Finally, the mRNA expression of target genes was measured by real-time PCR using 2 × Universal SYBR Green Fast qPCR Mix (Abclonal, Wuhan, China). The expression level of GAPDH was used as internal reference. The 2(−ΔΔCt) method was used to quantitatively calculate the expression level of the target genes. Primer sequences are provided in Additional file 1: Table S1.

### Statistic analysis

The data were presented as the mean ± SEM. Two-tailed Student's *t* test was used to compare the data between two groups. One-/two-way ANOVAs were used to examine significance among multiple groups, followed by Fisher’s least significant difference (LSD) post hoc test when groups were compared to each other. The data were analyzed by GraphPad Prism 9.0 or R software.

## Results

### Cognitive impairment and neuronal injury induced by Aβ_1–42_ i.c.v in mice.

To test the effect of Aβ_1–42_ i.c.v on the cognitive level of mice, we performed the Morris water maze test to investigate the cognitive level of mice in each group 4 weeks after surgery. During the five training days, the escape latency of platform searching was gradually decreased in each group, and the Aβ_1–42_ treatment can significantly extend escape latency compared with control and the sham-operated group (Fig. 1B). On the sixth day, we removed the hidden platform to detect the memory consolidation of each group. During the detection time of 60 s, the swimming time and distance of the Aβ_1–42_-treated group in the target quadrant were significantly reduced compared with the other two groups (Fig. 1C). In addition, Aβ_1–42_ treatment can decrease the mean crossing times and increased escape latency without changing the swimming distance and speed (Fig. 1D–G). Furthermore, the number of neurons in the hippocampus of each group was measured by immunofluorescence. Results showed that the number of neurons in the CA1, DG, and CA3 regions of the hippocampus of the Aβ_1–42_ treatment group was significantly reduced compared with the control group and the sham-operated group (Fig. 1H, I). In summary, Aβ_1–42_ can induce Alzheimer’s-like cognitive impairment and neuronal damage in mice.

### The diversity and composition of gut microbiota were changed after Aβ_1–42_ i.c.v for 4 weeks

**Fig. 2:**
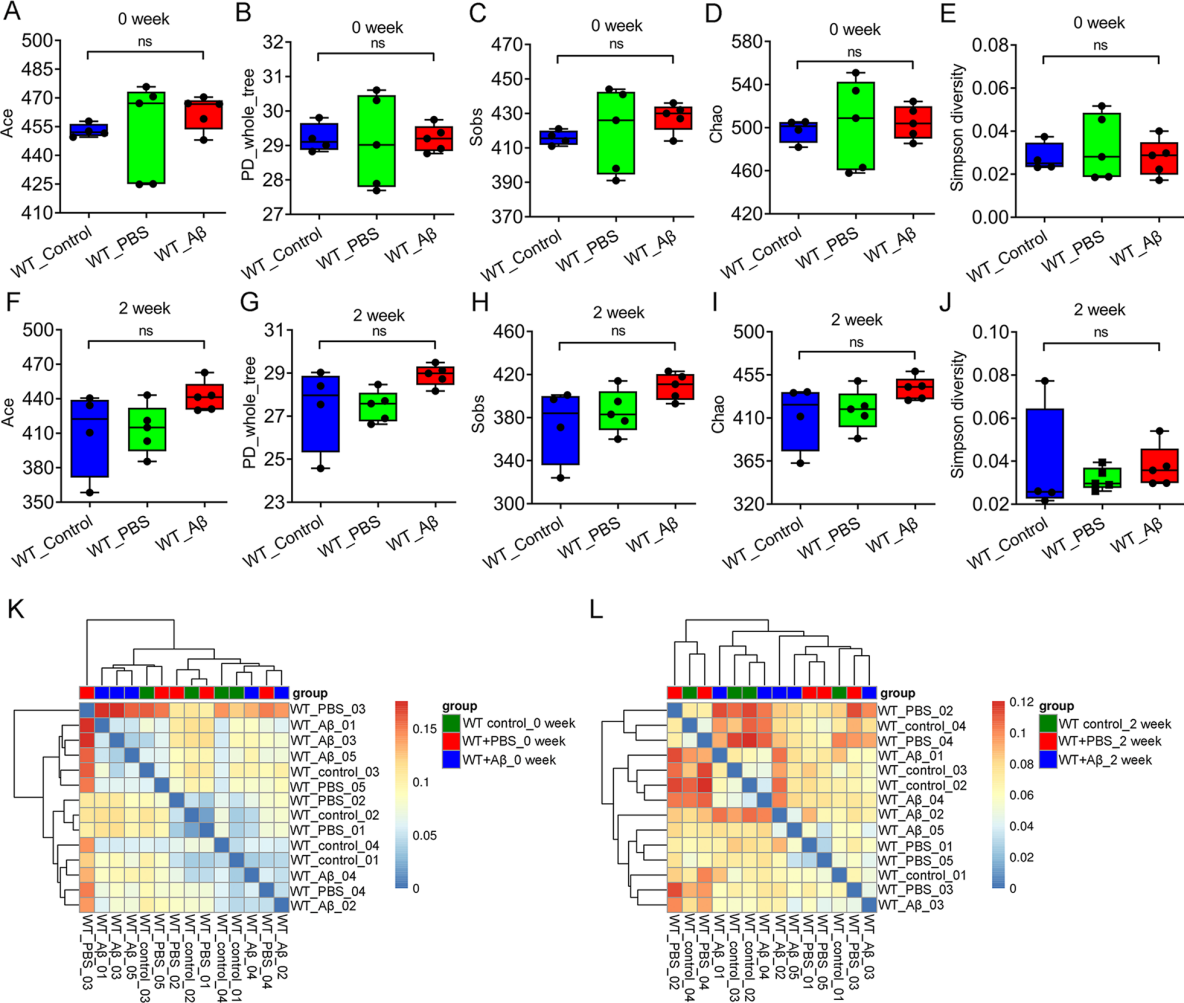
There was no statistically significant difference in the diversity of gut microbiota between baseline and 2 weeks after surgery among the three groups. **A**–**E** α diversity (Ace, PD whole tree, Sobs, Chao, and Simpson diversity) of gut microbiota at baseline in each group. **F**–**G** α diversity (Ace, PD whole tree, Sobs, Chao, and Simpson diversity) of gut microbiota in each group at 2 weeks after surgery. **K**, **L** Genus level of β diversity at baseline (left) and 2 weeks after surgery (right). n = 4–5 per group. Data were presented as mean ± SEM. Statistics were analyzed using one-way analysis of variance (ANOVA) and followed by Fisher’s least significant difference (LSD) test. *ns* no statistical difference

**Fig. 3:**
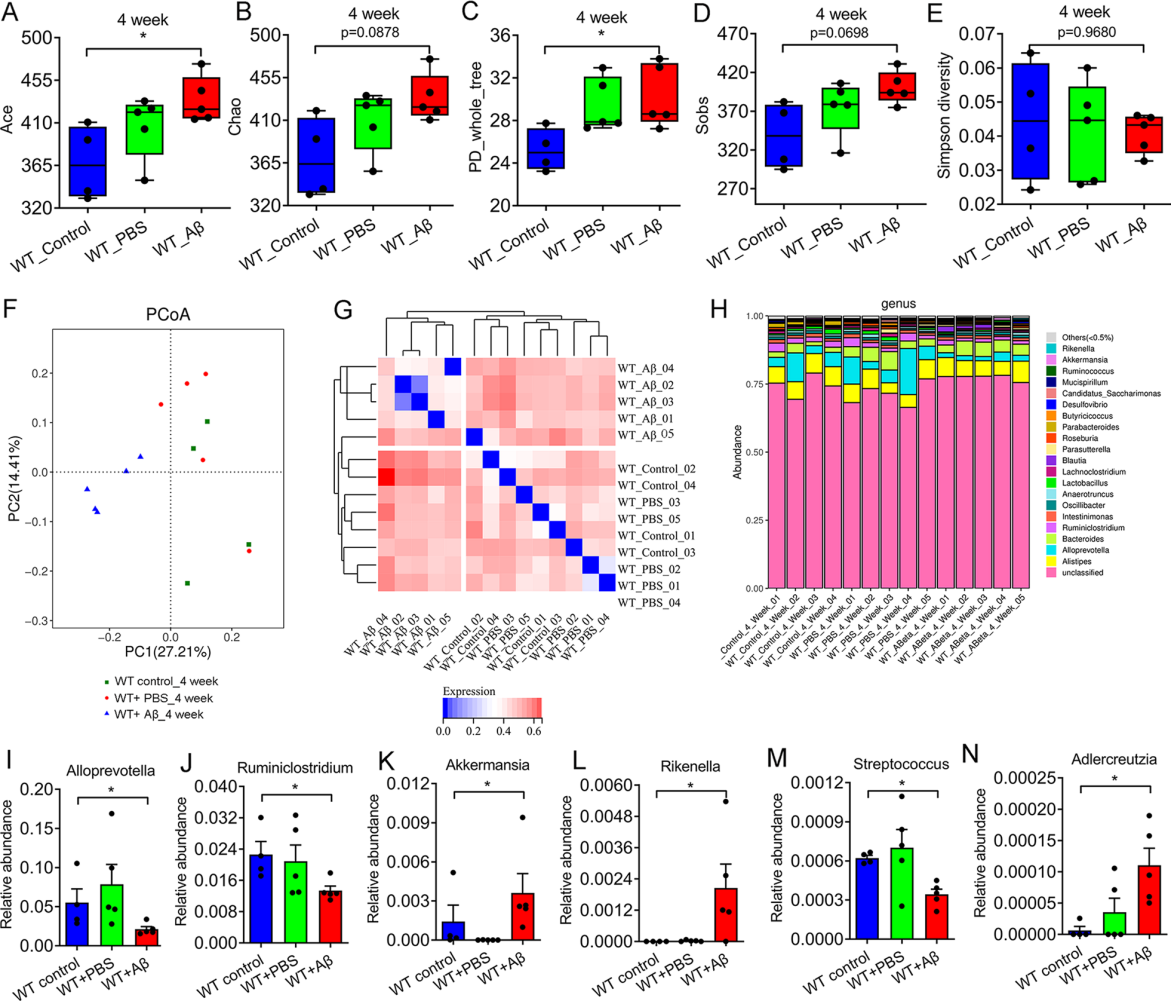
The diversity of gut microbiota was different in Aβ_1–42_ i.c.v group at the fourth week. **A**–**E** α diversity (Ace, PD whole tree, Sobs, Chao, and Simpson diversity) of gut microbiota at 4 weeks after surgery each group. **F** Principal co-ordinates analysis (PCoA) for β-diversity at genus level. **G** Clustering analysis for β-diversity at the genus level. **H** The composition of gut microbiota at the genus level. **I**–**N** Six bacterial genera had significant difference at the genus level among the three groups. n = 4–5 per group. Data were presented as mean ± SEM. Statistics were analyzed using one-way analysis of variance (ANOVA) and followed by Fisher’s least significant difference (LSD) test. ∗ P < 0.05, **P < 0.01

To investigate the effects of Aβ_1–42_ i.c.v on gut microbiota of mice, fecal samples of each group were collected before surgery, 2 weeks after surgery and 4 weeks after surgery for 16S rRNA detection. Our results showed that there was no significant difference in α and β diversity of the gut microbiota between the three groups at baseline and 2 weeks after surgery (Fig. 2). However, by the fourth week of surgery, there were statistically significant differences in α diversity of the gut microbiota between the three groups (Fig. 3A–E). In addition, β diversity results showed similar changes. At baseline and 2 weeks after surgery, β diversity of gut microbiota was not significantly separated in the three groups (Fig. 2K–L). By 4th week, the Aβ_1–42_-treated group were significantly separated with the control and the sham-operated group, suggesting Aβ_1–42_ treatment can significant change in the diversity of gut microbiota (Fig. 3F–G). We further analyzed the abundance of gut microbiota in each group at the genus level (Fig. 3H). The result showed that the abundance of *Alloprevotella*, *Ruminiclostridium*, and *Streptococcus* were decreased in Aβ_1–42_-treated group, compared with control and the sham-operated group (Fig. 3I, J, M). There were increased abundance of *Akkermansia*, *Rikenella*, and *Adlercreutia* in Aβ_1–42_ treatment group (Fig. 3K, L, N).

### Aβ_1–42_ i.c.v changed the body weight and colon structure of mice

**Fig. 4:**
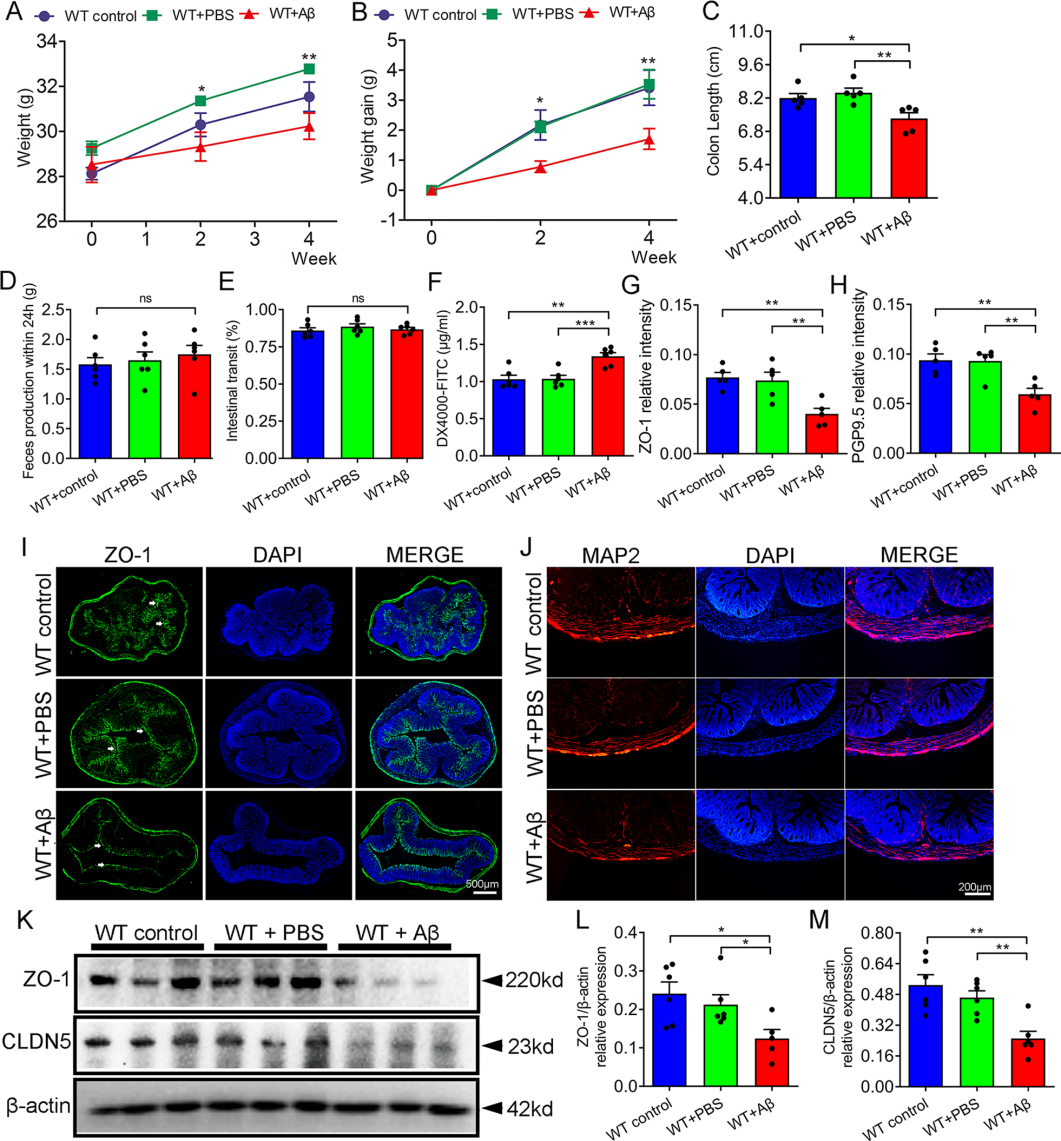
Aβ_1–42_ i.c.v induced changes of colonic structure in mice. **A**, **B** Weight and weight gain among three groups at baseline, 2 and 4 weeks after surgery. **C** Colon length of mice in each group 4 weeks after surgery. **D** Feces production within 24 h in each group 4 weeks after surgery. **E** Intestinal transit in each group 4 weeks after surgery. **F** Levels of serum FITC-dextran in each group 4 weeks after surgery 90 min after oral gavage. **G**, **I** Immunofluorescence and quantification of ZO-1 in colon of each group. Scale bars, 500 μm. **H**, **J** Immunofluorescence and quantification of PGP9.5 positive neurons in colon of each group. Scale bars, 200 μm. **K**–**M** Western blot and quantification of ZO-1 and CLDN5 in colon of each group. n = 6 per group. Data were presented as mean ± SEM. Statistics were analyzed using one-way analysis of variance (ANOVA) and followed by Fisher’s least significant difference (LSD) test. ∗ P < 0.05, **P < 0.01, *ns* no statistical difference

In this part, we further evaluated the gut structure and function of each group. First, we measured the body weight and the changes of body weight in each group at baseline, 2 and 4 weeks after operation. At baseline, there was no significant difference in body weight between the three groups (Fig. 4A). From the second week, the Aβ treatment group weighed less than other two groups (Fig. 4A, B). By the fourth week, the trend was even more pronounced (Fig. 4A, B). The colon length of Aβ-treated group was shorter than control and the sham-operated group (Fig. 4C). In addition, intestinal transit of each group was evaluated by blue Evans gavage and measuring feces production within 24 h. The results showed no difference of intestinal transit among the three groups (Fig. 4D–E). The intestinal permeability was assessed through the FITC-labelled dextran. There was a higher serum fluorescence values in Aβ-treated group than control and the sham-operated group. This suggested an increase in intestinal permeability in Aβ-treated group (Fig. 4F). We further detected the expression and distribution of intestinal barrier related protein: a major tight junction protein (ZO-1) by immunofluorescence and western blotting. Immunofluorescence staining in control and the sham-operated group displayed continuous distribution of ZO-1 at the colonic mucosa. In contrast, ZO-1 distribution in Aβ-treated group displayed as uneven distribution (Fig. 4G, I). The expression level of ZO-1 and CLDN5 protein was also significantly decreased in in Aβ-treated group, compared with other two groups (Fig. 4K–M). Intestinal function was regulated by intestinal neurons, so we further detected the distribution and number of neurons in the colon of each group of mice. The number of neurons in the colon of the Aβ-treated group was significantly reduced compared with that of control and the sham-operated group (Fig. 4H, J).

### Aβ_1–42_ i.c.v promoted gut inflammation through restraining cholinergic anti-inflammatory pathway

**Fig. 5:**
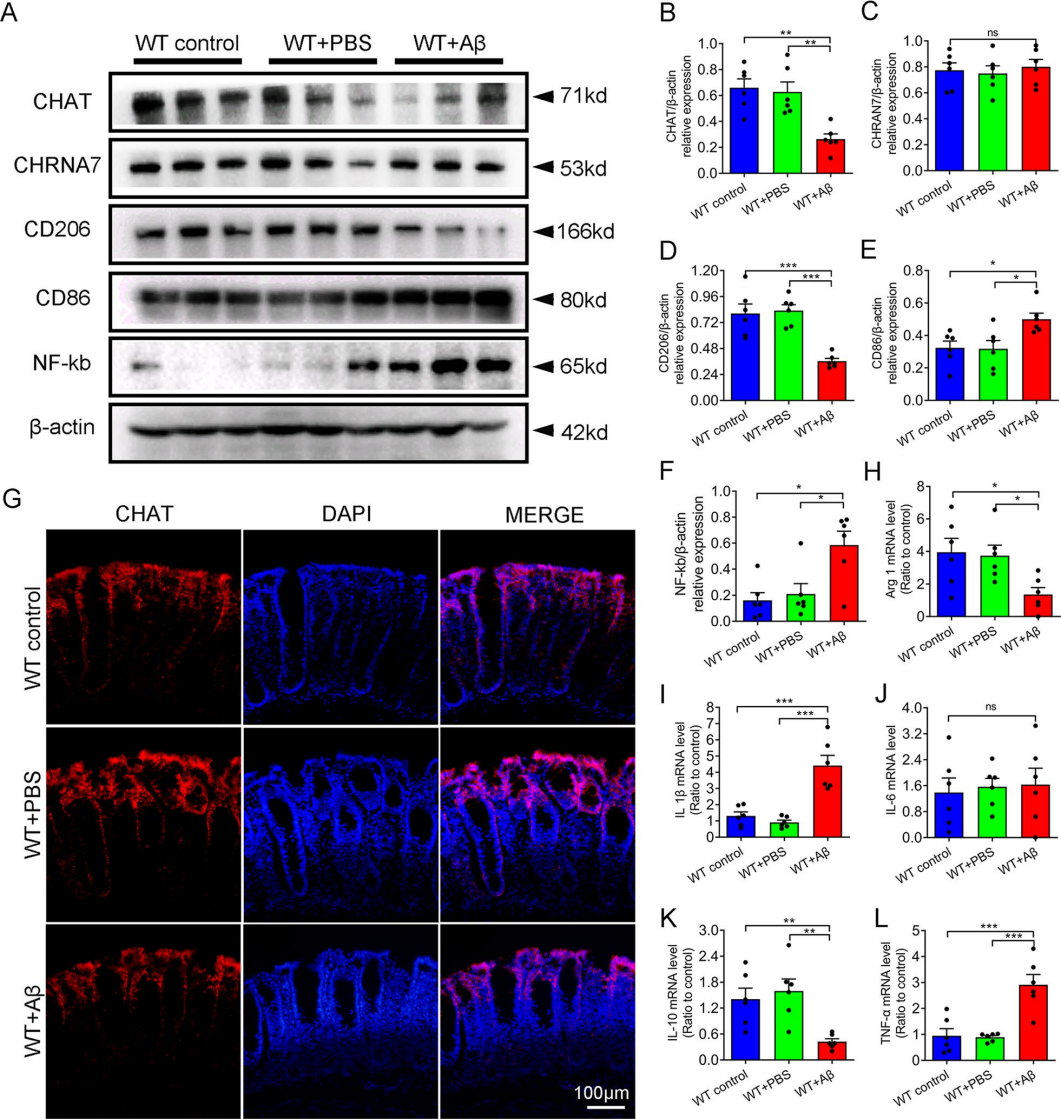
Aβ_1–42_ i.c.v inhibited cholinergic anti-inflammatory pathways and increased inflammation levels of colon. **A**–**F** Western blot and quantification of CHAT, CHRNA7, CD206, CD86, and NF-kb in colon of each group. **G** Immunofluorescence of CHAT in colon of each group. Scale bars, 100 μm. **H**–**L** Real-time PCR and quantification of inflammatory factor (TNF-α, IL-1β, IL-6, Arg-1, and IL-10) in colon of each group. n = 6 per group. Data were presented as mean ± SEM. Statistics were analyzed using one-way analysis of variance (ANOVA) and followed by Fisher’s least significant difference (LSD) test. ∗ P < 0.05, **P < 0.01, *ns* no statistical difference

As the most important parasympathetic nerve, vagus nerve was widely distributed in the digestive system. Vagus nerve-mediated cholinergic anti-inflammatory pathway was an important pathway for central nervous system to maintain intestinal homeostasis [28]. Therefore, we further explored whether altered intestinal homeostasis after Aβ_1–42_ administration was achieved through inhibition of cholinergic anti-inflammatory pathways. We examined the expression levels of surface markers “CD86” in pro-inflammatory macrophages and “CD206” anti-inflammatory macrophages. Increased CD86 expression was accompanied by decreased CD206 in the colon of Aβ_1–42_ administration group (Fig. 5A, D, E). In addition, the mRNA levels of pro-inflammatory cytokines TNF-α and IL-1β were significantly increased in the Aβ-treated group, while the levels of anti-inflammatory cytokine IL-10 were decreased (Fig. 5H–L). In addition, the expression level of choline acetyltransferase (CHAT) in Aβ-treated group was significantly decreased without the change of α7 subunit of the nicotinic ACh receptor (α7nAChR) (Fig. 5A–C, G), which suggested that Aβ intervention may inhibit the action of cholinergic anti-inflammatory pathways in the colon.

### Aβ_1–42_ i.c.v reduced M1 acetylcholine receptor expression  and promoted microglia activation

**Fig. 6:**
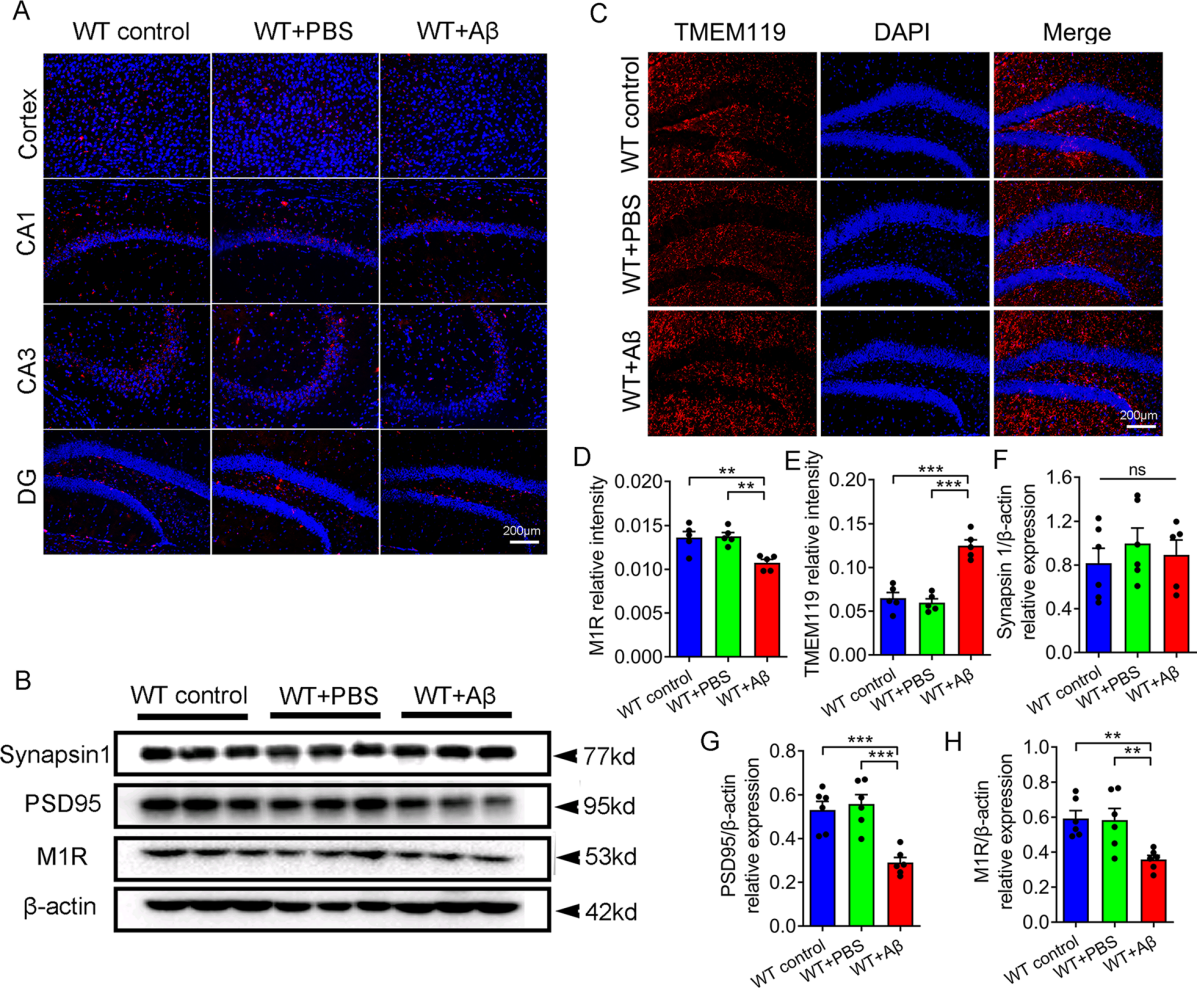
Aβ_1–42_ i.c.v reduced the M1 type acetylcholine receptors (M1 mAChR) expression in hippocampus and cortex. **A**, **D** Immunofluorescence and quantification of M1 mAChR in in hippocampus (CA1, CA3 and DG), and cortex of each group. Scale bars, 200 μm. **C**, **E** Immunofluorescence and quantification of TMEM119 in hippocampus DG of each group. Scale bars, 200 μm. **B**, **F**, **G**, **H** Western blot and quantification of synapsin 1, PSD95, and M1 mAChR in colon of each group. n = 6 per group. Data were presented as mean ± SEM. Statistics were analyzed using one-way analysis of variance (ANOVA) and followed by Fisher’s least significant difference (LSD) test. ∗ P < 0.05, **P < 0.01, *ns* no statistical difference

Previous studies have reported that the forebrain was identified as the advanced processing center of cholinergic anti-inflammatory pathway, which participated in peripheral regulation through M1 acetylcholine receptor [29, 30]. Therefore, we analyzed the expression and distribution of M1AChR in each group. The result showed that the expression level of M1AChR in forebrain and hippocampus of control group and sham operation group was significantly higher than that of Aβ_1–42_ treatment group (Fig. 6A, D). In addition, western blotting showed that the expression of M1R was decreased in the Aβ-treated group, along with the decreased expression of postsynaptic protein PSD95 (Fig. 6B, G, H). Presynaptic protein Synapsin1 showed no significant difference among the three groups (Fig. 6B, F). Furthermore, Aβ_1–42_ i.c.v can promote the proliferation and activation of microglia (Fig. 6C, E). In conclusion, Aβ_1–42_ ultimately inhibited the cholinergic anti-inflammatory pathway by promoting microglia activation and reducing M1AChR expression.

### Aβ_1–42_ i.c.v was involved in regulating brain and intestinal APP processing

**Fig. 7:**
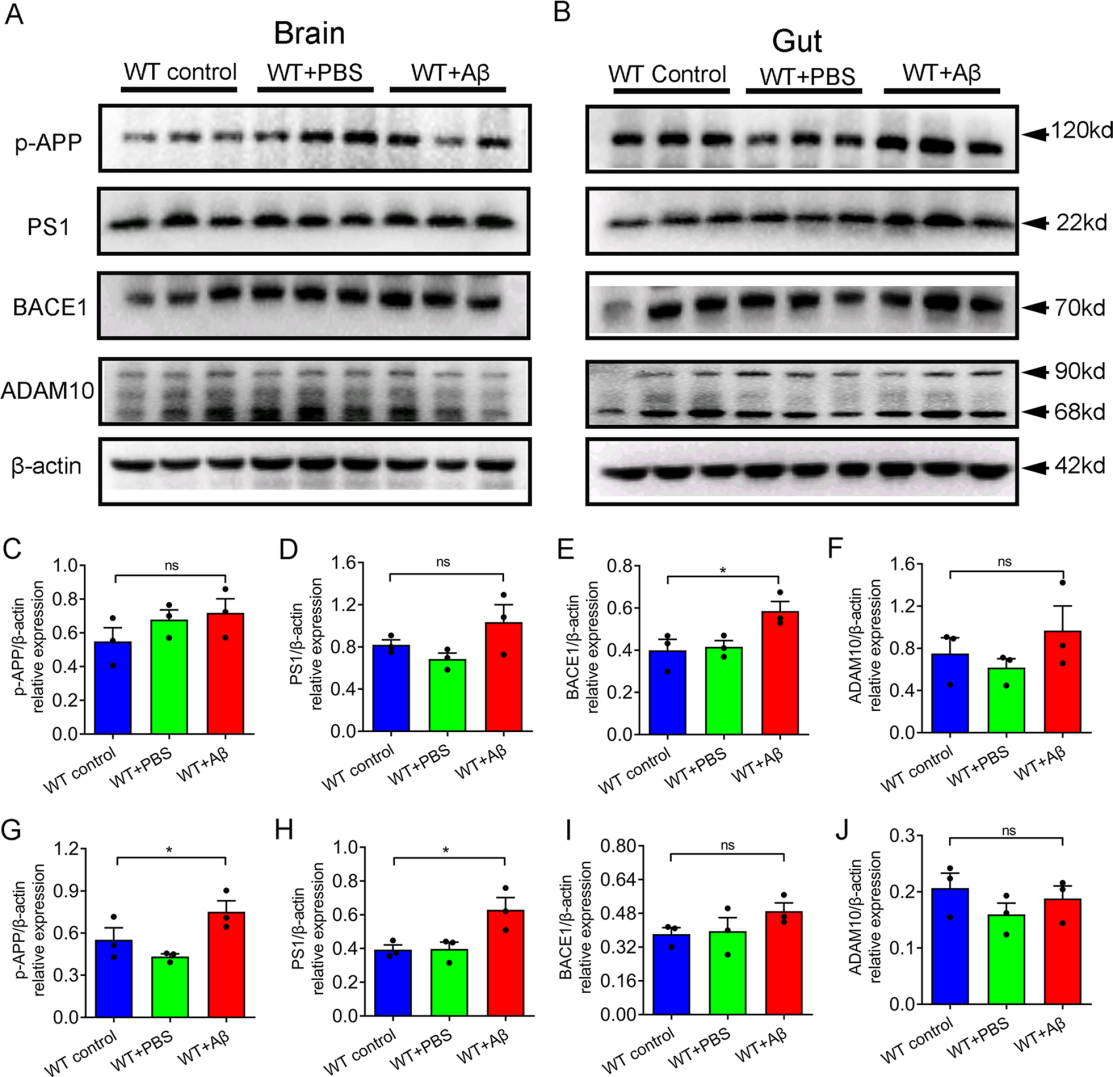
Aβ_1–42_ i.c.v accelerated the amyloidogenic pathways in brain and colon. **A**, **C**–**F** Western blot and quantification of p-APP, PS1, BACE1, and ADAM10) in brain of each group. (B, G-J) Western blot and quantification of p-APP, PS1, BACE1, and ADAM10) in colon of each group. n = 3 per group. Data were presented as mean ± SEM. Statistics were analyzed using one-way analysis of variance (ANOVA) and followed by Fisher’s least significant difference (LSD) test. ∗ P < 0.05, **P < 0.01, *ns* no statistical difference

Western blotting was used to investigate whether Aβ_1–42_ i.c.v was involved in the regulation of intestinal and brain APP process. In the brain, the expression level of BACE1 was significantly increased in the Aβ_1–42_-treated group, while the expression levels of p-APP, PS1 and ADAM10 were not statistically different among the three groups (Fig. 7A, C–F). In colon, Aβ_1–42_ i.c.v significantly increased the expression of p-APP and PS1, but without changing the expression of BACE and ADAM10 (Fig. 7B, G–J).

## Discussion

Alzheimer's disease, the most common type of dementia, has no clear pathogenesis. Recent studies have shown that there was dysbiosis of gut microbiota in AD patients and it correlated with the clinical and pathological features of AD [4]. More importantly, dysbiosis of the gut microbiota has been observed not only in AD patients, but also in patients with MCI patients [5]. In addition, the intervention strategy targeting gut microbiota was effective in delaying the course of AD [9]. However, the mechanism of gut microbiota dysbiosis in AD remains unclear.

In this study, we constructed AD model mice by injecting Aβ_1–42_ oligomers into the lateral ventricle, which was a widely adopted approach to building AD models [22–24]. The Morris water maze was the most widely used experiment to study spatial learning and memory in AD model mice [31]. Our behavioral results showed that the mice in the Aβ-treated group had worse learning and memory abilities than those in the control group and the sham-operated group. Furthermore, the number of neurons in the hippocampus of the Aβ_1–42_-treated group was significantly lower than that of the control group and the sham-operated group. These results indicated that AD model mice were successfully constructed by Aβ_1–42_ i.c.v in this study.

Gut microbiota difference analysis showed that there was no difference in α and β diversity of gut microbiota between the three groups at baseline. After 2 weeks of treatment, although no differences in α and β diversity were observed between the three groups, a trend of differences was observed. By four weeks after surgery, there were significantly differences in α-diversity (Ace and PD whole tree) among the three groups. In addition, the β diversity of Aβ-treated group was significantly separated from that of control group and sham operation group. These results suggested that Aβ_1–42_ i.c.v can cause the alteration in the abundance of gut microbiota. In AD rat model caused by Aβ_1–42_ i.c.v, Xu et al. reported that Aβ injection could also cause the alteration in gut microflora at 4 weeks, which was consistent with our results [32]. In the analysis of gut microbiota abundance at the genus level, we found a decrease in the abundance of *Alloprevotella*, *Ruminiclostridium*, and *Streptococcus*, and an increase in the abundance of *Akkermansia*, *Rikenella*, and *Adlercreutzia* in the Aβ-treated group. Previous studies reported that *Akkermansia* abundance was increased in APP/PS1 mice and the abundance of *Rikenella* was upregulated in Aβ_1–42_-induced mice [33, 34]. In addition, there was a decreased abundance of *Ruminiclostridium* in AD patients [35]*.* These results were consistent with our report. In 2021, Kamble et al. found that neprilysin enzyme from *Streptococcus suis GZ1* can take part in cleave Aβ peptide [36]. This may explain the reduced abundance of *Adlercreutzia* in the Aβ-treated group. Furthermore, Aβ_1–42_ i.c.v can induce changes in intestinal structure, including colon length, intestinal mucosal barrier integrity, and enteric neuron number in mice. Previous studies have shown similar changes in intestinal structure and function in transgenic AD model mice [8, 37]. For example, Puig et al. reported that occludin, an epithelial tight junction protein, were overall decreased in small intestine of APP/PS1 mice [8]. In addition to the changes of gut anatomy and motility, there were also elongated phenotypes of primary cilia in 5xFAD mice [37].

The vagus nerve, an important bridge between the CNS and gut, can transmit the information of the periphery to the CNS, and feedback the regulating effect of the CNS to the periphery. Cholinergic anti-inflammatory pathway is a vagus nerve-mediated signaling pathway that the CNS regulates peripheral immune system homeostasis [38]. In the CNS, the cholinergic anti-inflammatory pathway is regulated by the M1 type acetylcholine receptors, which is widely expressed in the cortex and hippocampus [39]. Several previous studies have suggested that activating cholinergic anti-inflammatory pathways was a potential treatment for AD [40, 41]. For example, the administration of acetylcholinesterase inhibitors (AChEIs) can increase acetylcholine levels to provide neuroprotection, reduce Aβ fibrils toxicity, and regulate inflammatory response [40]. The positive allosteric modulator of α7 nAChR can improve cognitive function of APP/PS1 mice through promoting the anti-inflammatory effect [42]. In peripheral, activated efferent fibers of the vagus nerve release acetylcholine. The Ach can activate the α7 acetylcholine receptor (α7 nAChR) on the surface of macrophages to induce the transformation of macrophages from a pro-inflammatory to an anti-inflammatory form through inhibiting NF-kb pathway [17]. In this study, Aβ_1–42_ treatment can reduce the expression of M1 mAChR in hippocampus and cortex, which was also markedly decreased in AD patients’ brain [43]. In colon, we detected that treatment with Aβ_1–42_ reduced the expression level of colonic acetylcholine transferase, the only synthetase for Ach [38]. In addition, Aβ_1–42_ treatment reduced the expression level of anti-inflammatory macrophage markers (CD206 ) and increased the expression level of pro-inflammatory markers (CD86) in colon. At the same time, an increase in proinflammatory factors (TNF-α, IL-1β, and IL-6) and a decrease in anti-inflammatory factors (IL-10) were detected in the Aβ treatment group. These results consistently suggested that the cholinergic anti-inflammatory pathway was impaired after Aβ_1–42_ i.c.v. In APP/PS1 mice, an increase in pro-inflammatory factors and activating immune cells was also detected in small intestine [8]. A recent study showed that mice with Chrna7 gene knockout mice showed depression-like behavior and gut microbiota dysbiosis [44]. Based on the above results, we speculated that the gut microbiota dysbiosis induced by Aβ_1–42_ i.c.v. was closely related to the inhibition of cholinergic anti-inflammatory pathway.

Amyloid-β plaques formed by extracellular accumulation of Aβ are one of the important pathological features of AD. Aβ is derived by β and γ secretase hydrolysis of the β-amyloid precursor protein (APP), which is called the amyloidogenic pathways [45]. In the anti-amyloidogenic pathways, the APP is produced by the consecutive action of α and γ secretase [45]. In this study, we demonstrated that Aβ_1–42_ treatment can not only promote the amyloidogenic pathways through upregulating the expression of BACE1 in the brain, but also accelerate the amyloidogenic pathways through increasing the phosphorylation of APP and PS1 expression in the gut. In APP/PS1 mice, the amyloidogenic pathways was also upregulated in the small intestine [8]. Recent studies have shown that Aβ_1–42_ oligomers in the intestinal tract can be transferred into the brain by vagus nerve to induce AD pathology [46].

There were several limitations in this study. First, due to the difficulty of selective separation of vagus efferent fibers, we did not isolate the vagus efferent fibers of mice in each group to directly prove the role of vagus efferent fibers. Secondly, we only demonstrated that the cholinergic anti-inflammatory pathway inhibited by Aβ_1–42_ i.c.v was associated with gut microbiota dysbiosis. In future studies, we will further explore the direct effect between them. In addition, we will explore the association between gut microbiota dysbiosis and cholinergic anti-inflammatory pathways in AD patients.

## Conclusions

**Fig. 8:**
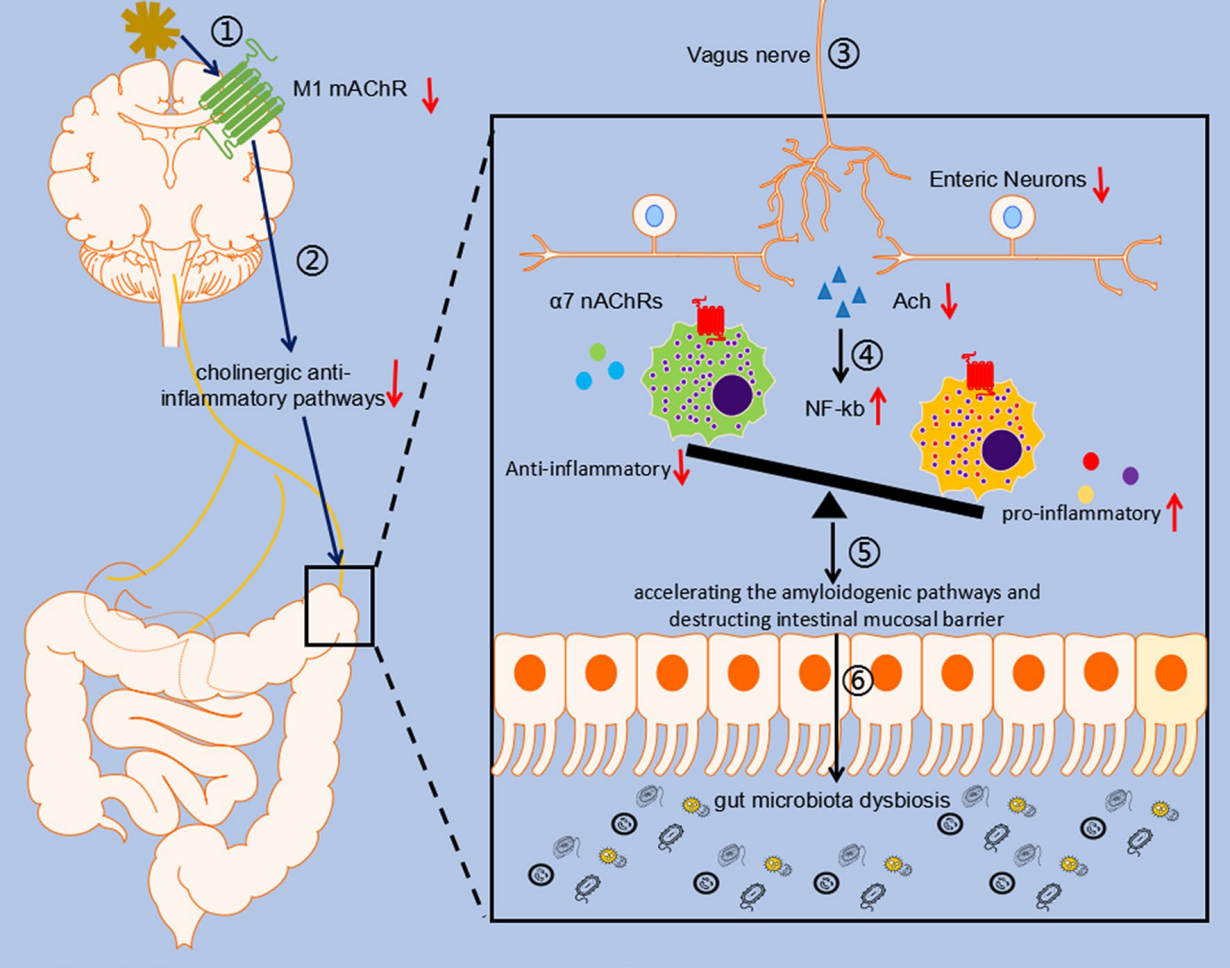
Diagram of the hypothesis model in this study. In Alzheimer’s disease (AD), Aβ can inhibit the vagus nerve-mediated cholinergic anti-inflammatory pathway through reducing the expression level of M1 type acetylcholine receptors (M1 mAChR) in the brain. In the gut, inhibition of the cholinergic anti-inflammatory pathway leads to decreased acetylcholine transferase (CHAT) levels, which reduces acetylcholine (Ach) secretion. As decreased Ach, the activation of α7 acetylcholine receptor (α7 nAChR), a receptor of Ach on the surface of macrophages in the intestine, is reduced. Then, the macrophages shift more toward pro-inflammatory phenotype by upregulating the NF-kb signaling pathway, leading to damage of enteric neurons, disruption of the intestinal mucosal barrier, and promotion the amyloidogenic pathways in the gut. Finally, gut microbiota dysbiosis occurs in AD

This study explained the law and mechanism of inducing changes in gut microbiota and intestinal function in AD (Fig. 8). First, the changes in gut microbiota caused by Aβ_1–42_ intervention were gradual process, accompanied by changes in intestinal structure. Second, the vagus nerve mediated cholinergic anti-inflammatory pathway was an important mechanism in the CNS for regulating intestinal homeostasis, which can be impaired by Aβ in AD. Finally, Aβ_1–42_ i.c.v can not only affect the APP processing in the brain, but also in the intestine to promote the production of Aβ. The imbalance of gut microbiota, intestinal immune system dysfunction and amyloidogenic pathways activation caused by Aβ_1–42_ i.c.v may further feed back into the CNS to accelerate the pathological process of AD.

## Supplementary Information

**Additional file 1:** **Table S1**: Primer sequences

## Data Availability

Data and materials will be shared upon reasonable request.

## Abbreviations

AD: Alzheimer's disease
MCI: Mild cognitive impairment
CNS: Central nervous system
AChR: Acetylcholine receptor
CHAT: Choline acetyltransferase
α7nAChR: α7-Subunit of the nicotinic ACh receptor
M1AChR: M1 acetylcholine receptor
APP: β-Amyloid precursor protein
i.c.v: Intracerebroventricular injection
