# Supplementary material for: Injection of amyloid-β to lateral ventricle induces gut microbiota dysbiosis in association with inhibition of cholinergic anti-inflammatory pathways in Alzheimer’s disease
Source: J Neuroinflammation. 2022 Sep 28;19:236. doi: 10.1186/s12974-022-02599-4 (PMC9520842; doi:10.1186/s12974-022-02599-4)
Supplement: Supplementary file 1 — Additional file 1: Table S1: Primer sequences [file 12974_2022_2599_MOESM1_ESM.docx]

**Supplementary Table 1**

**Table S1: Primer sequences**

| **Genes** | **Primer sequence (5’-3’)** |
| --- | --- |
| GAPDH | FP: TGTTCCTACCCCCAATGTGT |
|  | RP: CCCTGTTGCTGTAGCCGTAT |
| TNF-α | FP: TGGCCTCCCTCTATCAGTT |
|  | RP: GCTTGTCACTCGAAATTTGAGAAG |
| IL-1β | FP: TGGTGTGTGACGTTCCCATT |
|  | RP: CAGCACGAGGGTTTTTTGTTG |
| IL-6 | FP: TAGTCCTTCCTACCCCAATTTCC |
|  | RP: TTGGTCCTTAGCCACTCCTTC |
| IL-10 | FP: GCCAGAGCCACATGCTCCTA |
|  | RP: GATAAGGCTTGGCAACCCAAGTAA |
| Arg-1 | FP: TTGGCAAGGTGATGGAAGAGACCT |
|  | RP: CGAAGCAAGCCAAGGTTAAAGCCA |

Note:

FP: forward primer; RP: reverse primer.
